# The first report of a known 4A syndrome patient with suspected manifestations of COVID-19, what was the final outcome?

**DOI:** 10.1016/j.heliyon.2022.e11766

**Published:** 2022-11-19

**Authors:** Elham Azmoodeh, Amirhessam Kheirieh, Sepideh Mahdavi, Fatemeh Toufan, Sepideh Nazemi

**Affiliations:** a School of Medicine, Shahroud University of Medical Sciences, Shahroud, Iran; b Department of Epidemiology, School of Public Health, Iran University of Medical Sciences, Tehran, Iran; c Department of Internal Medicine, Clinical Research Development Unit, Bahar Hospital, Shahroud University of Medical Sciences, Shahroud, Iran; d Department of Internal Medicine, Clinical Research Development Unit, Imam Hossein Hospital, Shahroud University of Medical Sciences, Shahroud, Iran

**Keywords:** 4A syndrome, Addison’s disease, Alacrima, Achalasia, COVID-19

## Abstract

The present study was performed on a 24-year-old Iranian man referred to Hospital with suspected symptoms of COVID-19, including fever, weakness, and cough. According to medical history, he had Alacrima, esophageal Achalasia, and adrenal insufficiency from childhood.

Based on medical records and clinical examinations, the physician suspected 3A syndrome in the patient and requested further examination for MRI, CXR, and COVID-19 RT-PCR test. The result of the COVID-19 RT-PCR test was negative the next day. The patient’s CXR showed ground-glass opacity (GGO) and pulmonary fibrosis. Based on images and MRI reports, severe posterior cortical atrophy disproportionate to chronological age and bilateral atrophy of the lacrimal gland were reported. After reviewing and summarizing the records, history, examinations, and Paraclinical tests, the patient was identified as a case of 4A syndrome.

## Highlights

• Syndrome 4A is characterized in increasing order by manifestations of lack of tears, vomiting, hypoglycemia, and finally, lack of pupil dilation and severe sweating from infancy to youth.
• Usually, the first sign of suspicion of a genetic defect in the AAAS gene is the lack of tears that appears since infancy.
• It seems that a protected lifestyle and prevention of infection in affected people is the best way to control and adapt to this syndrome.
• Due to frequent infections, especially various pneumonias, these people are at high risk of contracting COVID-19, so completing vaccinations and following health protocols are vital and necessary.

## 1 Introduction

4A syndrome is a multi-system disease characterized by Addison’s disease, autonomic nervous system disorders, Alacrima, and Achalasia [1]. In some cases, if the disease is diagnosed at an early age, there might be no autonomic nervous system disorder, so it would be called AAA syndrome or Allgrove syndrome, first introduced in 1978 [2]. This syndrome is one of the rare genetic diseases that affects approximately one in a million people, even though this estimation may be lower than the actual rate due to lack of diagnosis [3]. Most disease cases have been reported to be blacks, Arabs, Native Americans, and Asians [4]. In addition to the three main symptoms that characterize this disease, patients may also have neurological and dermatological manifestations or have different phenotypes due to microcephaly and short stature [5, 6]. Genetic studies indicate that in this syndrome, about 90% of mutations are in the form of AAAS gene replication on chromosome 12q13, and in most cases, this autosomal recessive disease occurs with a negative family history [3, 7]. The AAAS gene is involved in the production of the 546 amino acid polypeptides called ALADIN protein, which is responsible for the homeostasis of adrenal cells and thus inhibits steroidogenesis so that high expression of this protein is observed in the adrenal glands, nervous system, and gastrointestinal tract; The same organs in which the main pathological manifestations of the disease occur [8, 9].

In this article, we will introduce a 24-year-old patient who had referred with the initial symptoms of COVID-19. after examination, it was found that he has some important past medical history such as Alacrima, Achalasia, Addison's disease, and autonomic nervous system disorders, which had not been known case as 4A syndrome until now. At the end, we will also discuss the complications of this disease and final outcome of suspected COVID-19 alongside it.

## 2 Case report

The patient was a 24-year-old Iranian single man who was 160 cm tall, 36 kg fat with BMI = 14, referred to Bahar Hospital with suspected symptoms of COVID-19, including low-grade fever, weakness and fatigue, and cough. At the specialist’s first visit, the specialist was attracted by the patient’s history and clinical signs. He announces that his symptoms have started six days before going to the hospital. The patient did not give us a history of COVID-19 vaccination, smoking, alcohol, or drug abuse, and he did not give a history of recent travel or contact with patients infected with COVID-19 or animals.

According to the patient, he has been diagnosed with Alacrima disease in infancy, Achalasia at age 4 (2002), and adrenal insufficiency at age 9 (2011). He has been hospitalized several times due to digestive problems; He also had a history of recurrent episodes of respiratory infection and productive cough, leading to TB diagnosis and hospitalization. The patient had no tears from birth, and after each breastfeeding, he experienced projectile vomiting containing breast milk; therefore, he has been examined and diagnosed with Achalasia. Besides, hypoglycemia attacks were diagnosed during his childhood due to severe darkening of the skin and gums, and he was treated for primary adrenal insufficiency with medicine.

**Figure 1:**
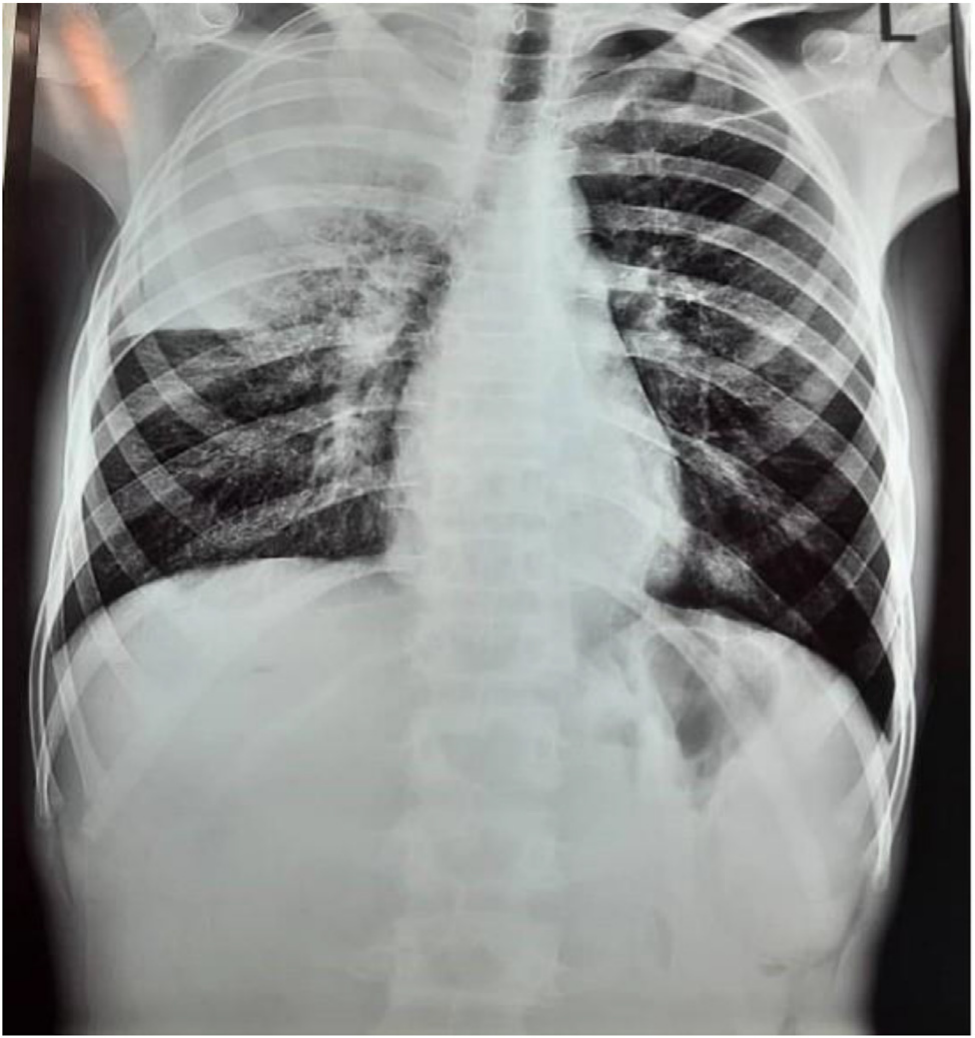
Chest X-Ray revealed Consolidation in right upper lob. Most probably due to local Pneumonia.

**Figure 2:**
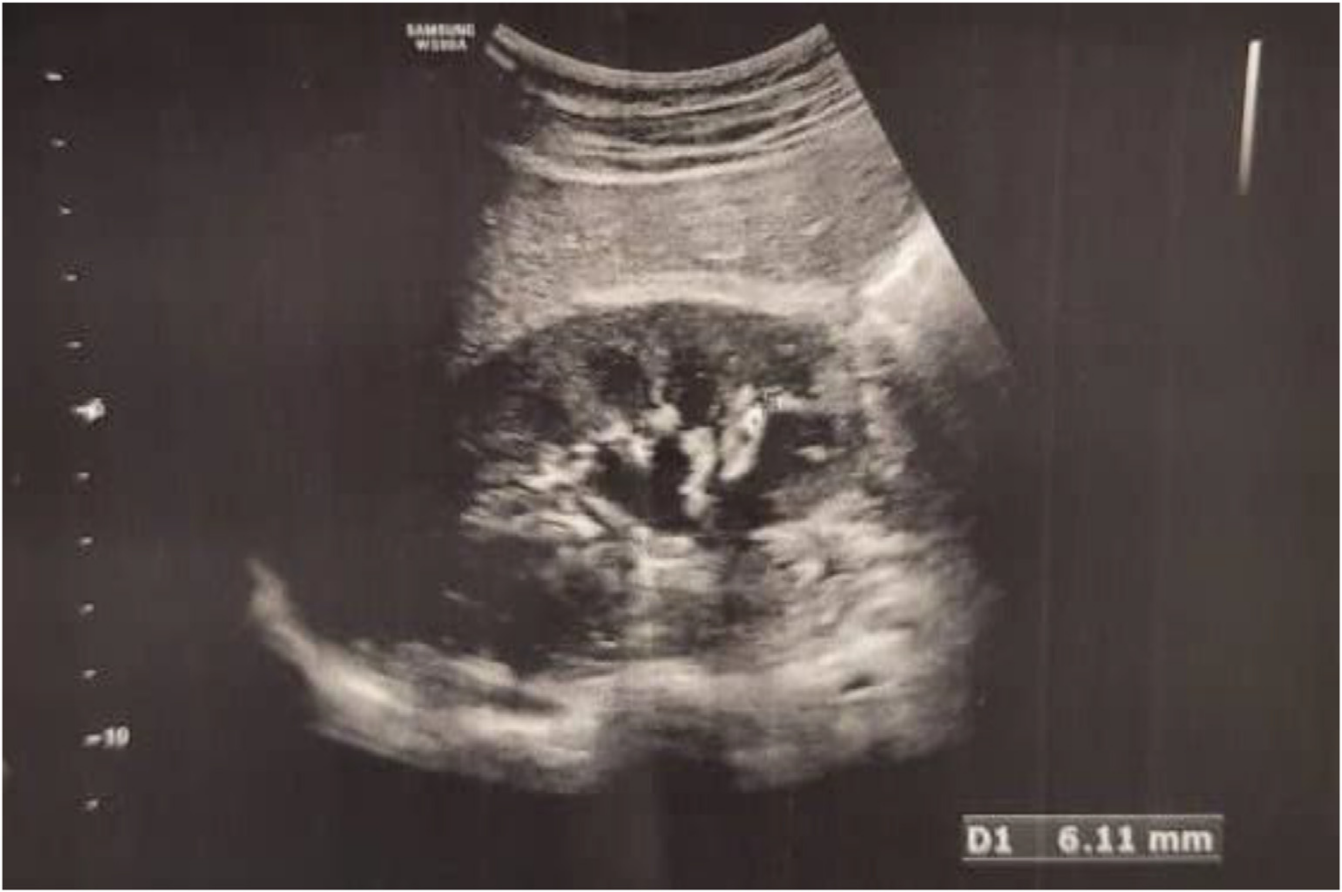
Kidney ultrasonography: Mild dilatation of pyelocaliceal system.

The following points are also noteworthy in the patient’s medical history. He had several dilatations and esophageal myotomy due to Achalasia at 4, 6, and 8. When he was eight years old (2006), the patient visited a physician due to fatty diarrhea and was treated after confirming the presence of a cyst of Giardia in his feces. When he was nine years old (2007), he was diagnosed with adrenal insufficiency (Addison’s disease) due to hypoglycemic attacks occurring at school. The patient also mentioned a history of several hospitalizations for pneumonia, which first occurred when he was 15 in 2013 (Figure 1), three years later (2016), and five years later (2018). According to the patient himself, his worst hospitalization and illness experience was due to pulmonary tuberculosis at the age of 16. At this age, the patient visits a doctor due to hemoptysis, and after three culture media were positive and BAL and CXR were performed, the patient’s pulmonary tuberculosis was confirmed and treated for six months. One year later, the patient was diagnosed with hypertensive crisis along with headache and dizziness for the first time, and after receiving the appropriate medication and re-determining the dose of the medication, he was discharged. In the same year, he underwent an ultrasound due to colicky pains in the sides, and a slight dilation was observed in the pilocalix system (Figure 2).

In addition to complaints and hospitalizations that have always imposed a psychological and financial burden on the patient, osteoporosis was also causing difficulties for him. He also experienced a femoral fracture resulting from a minor motorcycle accident two months ago, in March 2021.

Currently, the patient’s medications include Fluconazole capsule 150 mg PO daily, Tab Hydrocortisone 15 mg PO daily, Pearl Vitamin D3 50000 IU PO monthly, Calcium Carbonate tablet PO BD, artificial tear eye two drops. He was diagnosed with osteoporosis three years ago, and he was treated with Bisphosphonates weekly. However, he has recently been treated with Zoledronic acid injection annually. Also, Losartan tablet 25 mg PO daily should be taken periodically and under the physician’s direction when periodic hypertension occurs and should be stopped after eliminating the disease.

According to family records, the patient came from a consanguineous marriage and has four older brothers and sisters, all genetically healthy and have not had a known disease till now. Clinical examination showed that the patient had a mild fever, cough, anisocoria, joint hypermobility, decreased force of proximal muscles of the lower extremities and atrophic muscles, especially in the limbs, hyperkeratosis of the palms, hyperpigmentation on the extensor surface of the MCP joints, obvious dry skin, dryness and redness of the eyes, dry mouth and bilateral oral thrush. Due to severe dental infections, all the patient’s teeth were extracted two years ago in 2019. The patient had body dysmorphic disorder, a low tone, and nasal speech. The patient also had a history of occasional colicky pain and painful urination in the past episodically, which is not a problem now. He also mentions severe sweating during the day leading to his malfunction.

According to the patient’s history and symptoms, the RT-PCR test was requested and performed on May 22, 2021. Brian MRI without venous contrast and CXR were also required for the patient. The patient was discharged, but he was recommended to home quarantine, take fluids and return to the hospital in case of shortness of breath and high fever. The RT-PCR test result for COVID-19 reported in less than two days, and it was declared to be negative by the reference laboratory.

**Figure 3:**
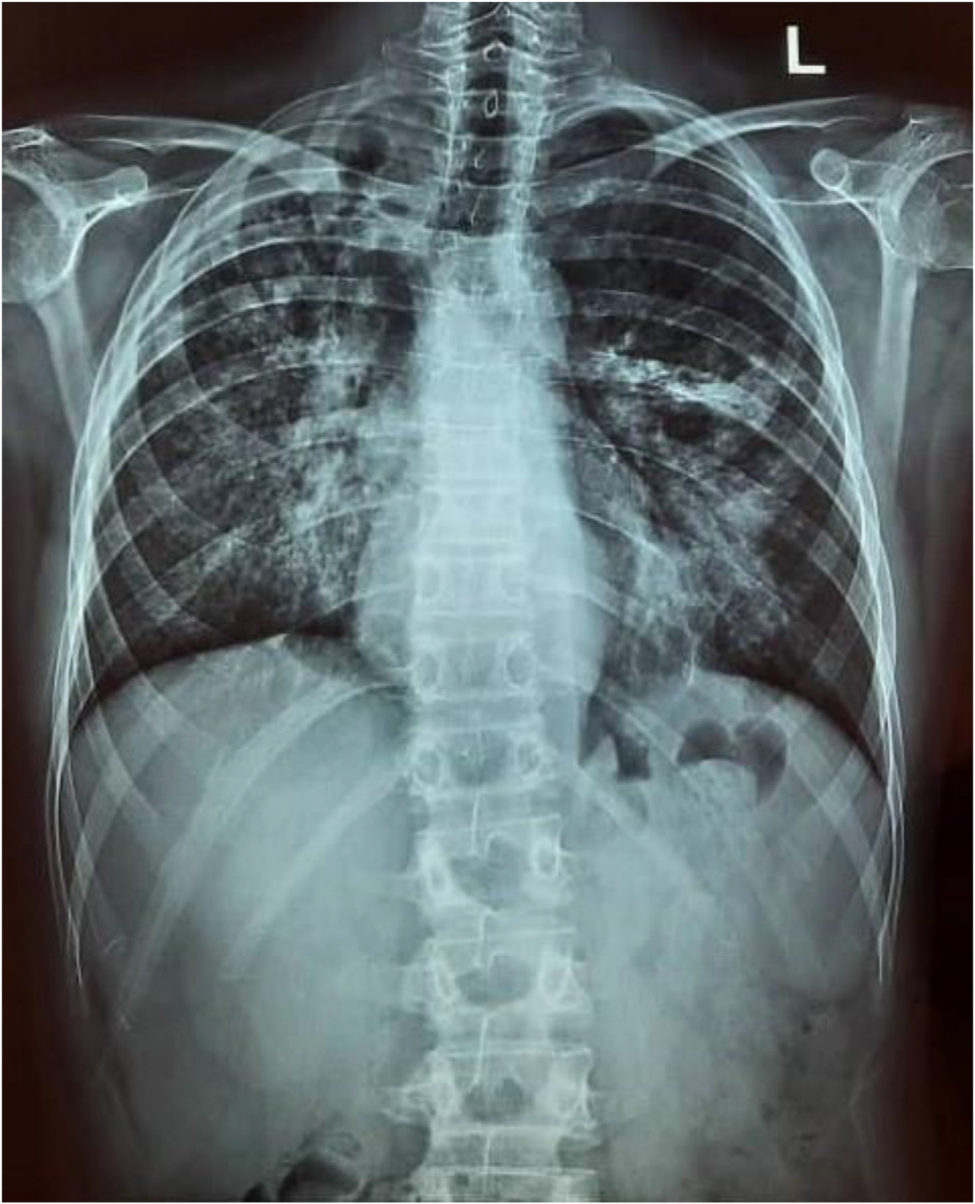
Chest X-Ray revealed Cavity with thick wall in right upper lobe along with the increase in thickness of pleura in the optic segment of the upper lob. Reticulonodular densities in the upper and middle zones of both sides and fibrosis, especially in the left middle zone, which are generally highly suggested for old TB.

**Figure 4:**
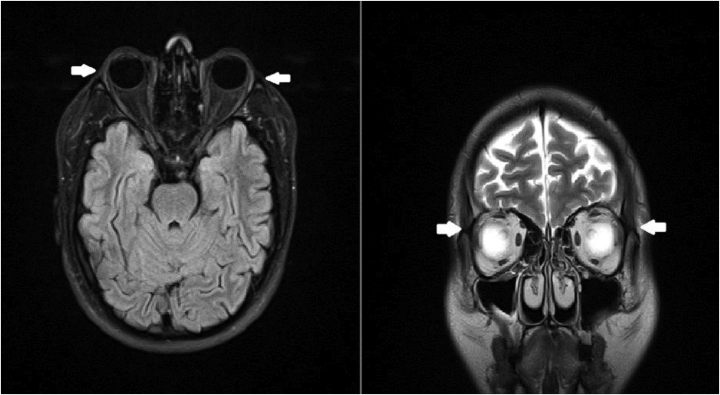
Brain MRI without contrast showing bilateral atrophic lacrimal glands with empty lacrimal fossa (white arrows).

The patient was re-examined by a physician two weeks later in June 2021. He followed the recommendations and became symptom-free with home treatment, despite long-term corticosteroid use from childhood, a history of pulmonary fibrosis, and his earlier symptoms. The initial symptoms of the patient, which were close to a viral infection of the upper respiratory tract, were cleared and final outcome of the disease was resolved without any problems or complications. Examining the patient’s CXR results reveals a ground-glass opacity and fibrosis, which may indicate the patient’s old pulmonary tuberculosis (Figure 3). Based on the images and MRI reports performed by a neurologist, severe cortical atrophy disproportionate to chronological age and bilateral lacrimal gland atrophy were reported (Figure 4).

Finally, after reviewing the patient’s history, studying the symptoms, MRI results, graphs performed, and summarizing all of the evidence, the specialist physician identifies and introduced the patient as a new case 4A syndrome for the first time.

## 3 Discussion

4A syndrome is a rare multi-system disease that varies in severity from person to person, with some being symptomatic in infancy and some in adolescence and adulthood, with more pronounced neurological problems [7]. The case proposed in this study weighed 36 kg and was 160 cm tall at the age of 24. Also, the patient’s nasal voice was clear while speaking, similar to the characteristics of the two cases introduced by Razavi et al. [10]. Children suffering from this disease generally have a different phenotype from healthy children, have a long and slender face and microcephaly, and are generally thin [11]. Typically, the first significant manifestation in these patients is Alacrima [12], which was also the first manifestation that our patient's parents noticed. Redness and dryness in adult eye examinations can also be a sign of Alacrima. It can be confirmed by the Schirmer’s test, which was not performed for our patient, but lacrimal gland atrophy was obvious on MRI results. Besides, lacrimal gland atrophy or agenesis was reported in other studies [13, 14].

Achalasia in patients with defects in the AAAS gene can be manifested from the age of 6 months to early adolescence [15], which can also coincide with the onset of complementary feeding in the child. However, in our case, Achalasia was manifested in infancy by vomiting, and despite dilatation and the Heller myotome, the patient still had dysphagia for solids. Dysphagia may not be improved in these patients due to the lack of saliva secretion [7]. Most patients also show adrenal insufficiency with hypoglycemic shock or hyperpigmentation symptoms. It is noteworthy that in primary adrenal insufficiency, the amount of potassium does not necessarily change and can be in the normal range [16], as it was in our patient. Therefore, we suggest that normal potassium levels in experiments do not lead to the rejection of adrenal insufficiency and that adequate and necessary examinations be performed because a common cause of death in these patients is primary adrenal insufficiency [6]. Osteoporosis at a young age is also another problem these patients reported in some cases [17, 18]. The bone density test in our patient also showed osteoporosis, which caused the lower limb fracture with mild trauma. Using glucocorticoids is not the only reason for osteoporosis in these patients, and several reasons, such as decreased physical activity and reduced use of sunlight in patients with severe neurological deficits, androgen deprivation, and poor nutrition, have also been identified [17]. Therefore, osteoporosis can be prevented by paying attention to physical activity and proper nutrition. Another important thing seen in our patients was the early loss of permanent teeth. As Razavi et al. pointed out in their study [10], it may be caused by reduced saliva and oral infections [19]. Gastroesophageal reflux disease in patients with Achalasia can also lead to tooth loss [20]. Hence, paying attention to oral health and making regular visits to the dentist is recommended.

According to previous studies, autonomic nervous system disorders occur in about 30% of patients with Triple A syndrome, in which case it is called 4A syndrome, and can have various manifestations such as hypotension or hypertension, decreased or increased sweating, cardiac arrhythmias, abnormal pupillary reflexes and anisocoria [6]. Our patient also had attacks of hypertension, increased sweating, and anisocoria. Neuroskeletal problems in these patients include hyperreflexia, gait disturbances, and decreased muscle tone and limb strength, which have also been observed in our patient. In the study of Kimber et al., these patients mentioned neurogenic degeneration in muscle biopsies, but its cause was unknown [21].

Another problem seen in these patients is the development of various infections that appear to be due to the long-term use of glucocorticoid drugs. For instance, in some patients, recurrent episodes of refractory pneumonia or pulmonary tuberculosis resistant to treatment and urinary tract infections, similar to ours, have been observed [22, 23, 24]. This patient’s disease was diagnosed because of the symptoms of upper respiratory tract infections suspected with COVID-19; however, despite long-term corticosteroid usage and the resulting weakened immune system and previous history of fibrosis, he did not develop any more severe or prolonged symptoms. His RT-PCR test was negative, and two weeks later, the patient was asymptomatic. Reviewing similar studies showed no other 4A patients who underwent COVID-19 examinations.

## 4 Conclusion

4A syndrome is one of the rare and multi-system diseases that causes many complications due to receiving long-term drugs. Since the first noticeable symptom is usually Alacrima in infancy, doctors should attempt to include this disease in their differential diagnoses as soon as Alacrima is confirmed and then take the necessary diagnostic measures. The prognosis of the disease largely depends on the early diagnosis of the disease, controlling the damage due to various infections, and determining the dose of drugs. It is recommended that physicians, knowing the progression of symptoms and with appropriate treatment, seek to reduce the complications of the disease and stop it as much as possible. Therefore, these patients must pay attention to their physical activity, proper nutrition, and oral hygiene. Due to neurological problems and adrenal insufficiency in these patients, which weakness the musculoskeletal system and immunity, COVID-19 can put these patients at serious risk, so it is recommended these patients be required to follow a protective lifestyle, such as wearing masks, protected sex, seasonal and COVID-19 vaccinations, etc., in order to prevent various infections.

## Declarations

### Author contribution statement

All authors listed have significantly contributed to the investigation, development and writing of this article.

### Funding statement

Sepideh Nazemi was supported by Shahroud University of Medical Sciences [99124].

### Data availability statement

Data included in article/supp. material/referenced in article.

### Declaration of interest’s statement

The authors declare no conflict of interest.

### Additional information

No additional information is available for this paper.
